# Influence of Hydration and Natural Carbonation Evolution on the Gas Permeability and Microstructure of Blended Cement Pastes

**DOI:** 10.3390/ma18184416

**Published:** 2025-09-22

**Authors:** Tomasz Tracz, Tomasz Zdeb, Krzysztof Witkowski, Daniel Szkotak

**Affiliations:** 1 Faculty of Civil Engineering, Cracow University of Technology, Warszawska Str. 24, 31-155 Kraków, Poland; tomasz.zdeb@pk.edu.pl; 2 Interdisciplinary Center for Circular Economy, Cracow University of Technology, Warszawska Str. 24, 31-155 Kraków, Poland; 3 Bruk-Bet Sp. z o.o., Nieciecza 199, 33-240 Żabno, Poland; krzysztof.witkowski@bruk-bet.pl (K.W.); daniel.szkotak@bruk-bet.pl (D.S.)

**Keywords:** cement paste, gas permeability, carbonation, hydration, microstructure

## Abstract

The high density of the internal structure of new-generation cementitious composites, such as high-performance and ultra-high-performance concretes, necessitates the use of advanced methods for evaluating their transport properties, particularly those employing a gaseous medium. The developed gas permeability method for cement pastes, based on a modified RILEM-Cembureau approach, has proven to be highly accurate, reliable, and extremely sensitive to changes in the porosity characteristics of such composites. The article contains the results of a study of the mass transport capabilities of blended cement pastes, characterised by variable water–cement ratios. Two types of cements were used in the study: with the addition of fly ash and blast furnace slag. Ordinary Portland cement was used as the reference binder. The tests were conducted after long-term curing under natural conditions, i.e., after 90 days and 2 years. The assessment of open porosity was carried out through three techniques: helium pycnometry, mercury intrusion porosimetry, and water saturation. Permeability, on the other hand, was measured using a customized approach tailored for uniform paste materials. Microstructural changes were also analysed in the context of natural hydration carbonation progress. The results presented allowed a quantitative description of the effects of the w/c ratio, the presence of additives, and the progress of hydration and carbonation on the porosity of pastes and their permeability to gas flow. The two-year curing period of the pastes exposed to natural CO_2_ resulted in a reduction of the permeability coefficient k ranging from 11% to 74%, depending on the type of cement and the water-to-cement (w/c) ratio. This decrease was caused by the continued progress of hydration and simultaneous carbonation. The results of the research presented are of interest from both an engineering and scientific point of view in the context of long-term microstructural changes and the mass transport abilities of cement pastes associated with these processes. The extensive range of materials compositions investigated makes it possible to analyse the durability and tightness of many cementitious composites over long periods of service.

## 1. Introduction

Standard methods are known for testing mass transport in cementitious composites, such as water absorption and watertightness tests performed in laboratory conditions [1,2,3,4,5] or in situ [6] or chloride ion diffusion tests [7,8,9]. All these methods are based on filling the pores of cementitious materials accessible to water, whose viscosity is about two orders of magnitude higher (~1 ×10^−3^ Pa·s) compared to gases such as CO_2_, SO_3_, or O_2_ (~0.01–0.02 × 10^−3^ Pa·s). The presence of these pores is relevant to the durability of concrete products and structures. According to [10], pores with diameters greater than 200 nm can only be completely filled with water at 100% humidity. When measuring the skeletal volume of solids using the gas method, i.e., helium pycnometry, He_(g)_ is believed to penetrate pores of less than 1 nm in diameter [11]. The assessment of transport capabilities using gas media is likely to be sensitive to the smallest changes in the pore structure of cement composites.

A well-known in situ method for evaluating the gas transport capacity of concrete is that developed by Torrent [12,13], where air is used as the medium. Based on Darcy’s law, the value of the permeability coefficient kT [m^2^] is determined, and where kT < 0.01 × 10^−16^ [m^2^], it is assumed that the concrete exhibits very good tightness, while where kT > 10 × 10^−16^ [m^2^], the concrete exhibits poor tightness [12].

As concerns laboratory testing, a fairly widely used method for testing gas permeability is the RILEM-Cembureau one [14], where oxygen, nitrogen or air are most often used as the medium. This method is most often used to evaluate the permeability of concrete. Similarly, as with the Torrent method, the value of the permeability coefficient k [m^2^] for stabilised gas flow is determined using Darcy’s law. Many researchers have confirmed the relationship between the two aforementioned methods [12,15]. Regardless of the chosen technique, employing inert gases in permeability testing offers the benefit of allowing repeated measurements without the complications typically associated with fluid interactions. These effects include leaching soluble components of the skeleton, hydration progress or shrinkage hysteresis caused by cyclic wetting and drying.

To date, studies of permeability to gas have mainly concerned concrete, a material with a highly heterogeneous structure. As a result, they demonstrate mass transport occurring simultaneously through interconnected open pores within the cement paste, the ITZ, and the aggregate, despite the fact that permeability across these regions can vary drastically, even by several orders of magnitude. In the interfacial transition zone, porosity is significantly increased due to the adsorption of water on the surface of unreacted aggregate grains, which leads to a local increase in the water–cement ratio in this area, and the uneven distribution of cement grains around the aggregate surface—the so-called wall effect. Thus, a local increase in the hydration degree of cement will occur, resulting in intensified binder contraction and consequently shrinkage as well as heterogeneous nucleation on the surface of aggregate grains of those phases for which the solubility product is exceeded, i.e., primarily Ca(OH)_2_. All these local variations in binder matrix uniformity increase the potential for composite mass transport and thus an increase in the kinetics of corrosion processes. According to Diamond’s research, for ordinary concrete with a w/c of 0.5, porosity in the ITZ can be as much as 50% higher than that observed for cement paste [16]. However, as porosity characteristics of concretes are largely dependent on the porosity of cement paste due to its volume share, it is precisely cement paste which, providing the continuous phase within these composites, is responsible for ensuring the required durability of concretes. Hence, the overarching goal of the research described in this article is to determine the gas permeability of cement pastes of varying compositions during different operation periods. In this study, a modified RILEM-Cembureau method was used, using an inert gas, i.e., nitrogen. The modification, which is described in detail in Section 2, was driven by the need to produce representative and homogeneous paste specimens free of shrinkage cracking, which is often observed especially at high w/c ratio values.

The impact of various material and environmental factors on changes in the open porosity of cement pastes has been the focus of interest of many researchers [17,18,19,20,21,22,23,24]. In [24], the authors determined the effect of the hydration time of cement pastes up to the 28th day of curing at varying water and cement proportions. They confirmed that the greatest changes in total open porosity, as determined using the MIP method, are observed until the 7th day of hydration, ranging from 5 to 8% depending on the value of the w/c ratio. More significant changes in the porosity described, in turn, are due to the composition of the paste, and primarily the amount of mixing water. An increase in the w/c ratio by 0.1 results in an increase in P_tot_ determined after 28 days of curing by approximately 5%.

While there is consensus about the effect of the degree of carbonation on the reduction in open porosity, and thus the transport capabilities of ordinary Portland cement pastes [25,26,27,28,29], in the case of blended cement pastes research results are inconclusive [27,28]. According to Morandeau et al. [26,30], the carbonation process causes a reduction in the porosity of cement pastes irrespective of the presence of SCMs (i.e., fly ash) dosed at up to 60%. Kurdowski [31] claims that all hardened cement paste ingredients, from hydrated calcium silicates, portlandite, Afm, and AFt to relic cement grains, are subject to carbonation according to Equations (1) to (5). Depending on its polymorphic variety, crystallised calcium carbonate can exhibit expansion or contraction relative to substrate volume [27,32]. According to the diagram published in [33], portlandite is the first compound to undergo carbonation. The depletion of calcium hydroxide in favour of predominantly calcite is associated with a sharp drop in the pH of the paste, which in turn leads to decalcification of the C-S-H phase. Further reaction with CO_2_ results in the sequential disappearance of monocarbonate, followed by ettringite, and finally hydrogarnets. The carbonation process itself does not appear to significantly affect the content of residual cement grains.

Ca(OH)_2_ + CO_2_ → CaCO_3_ + H_2_O (1)

1.7CaO·SiO_2_·2.5H_2_O + 1.7CO_2_ → 1.7CaCO_3_ + SiO_2 (am)_ + 2.5H_2_O (2)

4CaO·Al_2_O_3_·13H_2_O + 4CO_2_ → 4CaCO_3_ + 2Al(OH)_3_ + 10H_2_O (3)

3(3CaO·Al_2_O_3_·CaSO_4_·14H_2_O) + 6CO_2_ → 3CaO·Al_2_O_3_·3CaSO_4_·32H_2_O + 6CaCO_3_ + 2Al(OH)_3_ + 4H_2_O (4)

3CaO·Al_2_O_3_·3CaSO_4_·32H_2_O + 3CO_2_ → 3CaCO_3_ + 2Al(OH)_3_ + 3(CaSO_4_·2H_2_O) + 23H_2_O (5)

The portlandite carbonation mechanism as described by Swenson et al. [34] consists in the formation of a passivation layer consisting of calcium carbonate, which results in the entrapment of water released during the carbonation reaction. Material desiccation leads to a local moisture gradient forming, which results in vapour exerting pressure on the passivation layer, causing its subsequent destruction. The process of carbonation of hydrated calcium silicates is more complicated. In the first stage, decalcification occurs, which results in a decrease in the Ca/Si ratio and an increase in the degree of polymerisation of silicon-oxygen chains with a reduction in inter-packet spaces [35]. When the Ca/Si ratio equals 1.2, a reduction in the specific surface area of the C-S-H phase is observed. Moreover, once the Ca/Si ratio surpasses approximately 0.66, the phase undergoes full decomposition, leading to the breakdown of the cationic subnetwork formed by octahedral calcium-oxygen layers, and resulting in the formation of hydrated silica gel [36].

In the case of blended cement pastes, due to their reduced CH content, hydrated calcium silicates are more directly exposed to destruction caused by carbonation; however, due to their tighter structure, changes in permeability are not necessarily noticeable. The study results presented below focus on changes in the microstructure of blended cement pastes, and especially in their open porosity, which is the direct cause of the ability of cementitious composites to transport mass. A broad range of pastes which differed in terms of the type of cement used, i.e., cement with fly ash (FA) or ground granulated blast-furnace slag (GGBS) additions, and with different w/c ratios were studied.

The findings of research described below appear to hold value from both engineering and scientific point of view, particularly in understanding long-term microstructural evolution and the transport properties of cement pastes influenced by these transformations. By examining a wide range of material compositions, the study enables comprehensive assessment of the permeability of various cement-based composites over extended use. Furthermore, the insights provided are expected to support ongoing advances in testing techniques to assess the durability of contemporary cementitious materials.

## 2. Materials and Methods

### 2.1. Material and Specimen Preparation

The pastes analysed were made with pure Portland cement of strength class 42.5, consisting only of clinker and a gypsum setting time regulator; in order to obtain blended cements, OPC was replaced with 20% fly ash (FA) by weight in the first case and with 50% ground granulated blast furnace slag (GGBFS) by weight in the second case. Siliceous fly ash (FA) derived from the combustion of hard coal was used in this study. The material exhibited a high combined content of SiO_2_, Al_2_O_3_, and Fe_2_O_3_ (>88%), meeting the requirements of [37] for use as a supplementary cementitious material in cement and concrete production. The particle size distribution of FA, characterized by d_10_, d_50_, and d_90_ values, was 3.0 µm, 16.5 µm, and 38.0 µm, respectively. Ground granulated blast furnace slag (GGBFS) supplied by a certified cement manufacturer was also employed, conforming to the specifications of [38]. Its particle size distribution, defined by d_10_, d_50_, and d_90_, was 2.0 µm, 13.5 µm, and 26.5 µm, respectively. The proportions were chosen so as to approximate the compositions of the widely used Portland fly ash cement CEM II/A-V and blast furnace cement CEM III/A in accordance with European standard EN 197-1 [39]. As preliminary tests demonstrated, both binders produced in this manner met the requirements of strength class 42.5. The results of tests on the reference paste, i.e., produced from CEM I cement, were previously published in [40], but in order to facilitate comparative analysis with a binder that does not contain mineral additives, selected results are also included here. The characteristics of these ingredients and blended cements are presented in Table 1 and Table 2.

In order to conduct permeability tests of cement pastes using the RILEM-Cembureau method, it was necessary to reduce the size of specimens typically used for this type of testing. Numerous observations preceding the presented tests have shown that for cylindrical specimens with a diameter of 10 mm and a height of about 60 mm, the absence of shrinkage cracking and thus high repeatability of measurements could be achieved. The paste was formed in rigid PP tubes, which, once filled, were sealed with silicone stoppers to avoid exchange of mass with the external environment. During the initial curing period, i.e., for 28 days, the specimens were stored in a desiccator. After this period, the specimens were demoulded, and subsequently both ends were ground by about 5 mm to a height of 50 mm in order to eliminate potential inhomogeneities resulting from suspension sedimentation during the initial setting phase. After demoulding, the specimens were stored under laboratory conditions at a temperature of about 20 °C and a relative humidity of 60 ± 5% with the first group being tested after 90 days and the second only after 2 years to allow further progress of hydration, as well as natural carbonation. Prior to testing, the samples were dried at 40 °C until reaching a stable weight. This controlled drying temperature helped minimize alterations in the microstructure of the examined pastes [21].

Cement pastes were produced using varying amounts of mixing water so that the value of the water–cement ratio ranged from 0.3 to 0.6 with a variation of 0.1. Cement paste compositions are shown in Table 3. The mixing process of cement pastes was carried out according to the guidelines in EN 196-1 [41]. The range of w/c ratios was selected as to be representative of a wide range of concretes from ordinary through high-performance and highly liquid concretes, as well as dry concretes that require vibro-pressing compaction, for instance. For pastes with w/c ratios of 0.4 and 0.3, a small amount of superplasticiser was added to ensure that all pastes exhibited similar liquidity during moulding and thus to ensure comparable compaction of the suspension in PP tubes. Pastes with a w/c ratio of 0.6 were characterised by slight sedimentation, which was eliminated by the aforementioned grinding of the ends of the cylinders produced. Prior to microstructural testing, the specimens were subjected to slow drying to constant weight in a vacuum chamber at T = 40 °C and P = 20 mbar to avoid hydrate decomposition, rapid shrinkage and thus cracking that could significantly affect porosity distribution and the results of gas permeability measurements.

### 2.2. Methods

#### 2.2.1. Gas Permeability

The permeability of the pastes was tested using the RILEM-Cembureau method as described in [42,43]. The purpose of the study is to determine the permeability coefficient (k) for nitrogen flow according to the following equation.

(6) $$k=\frac{2QP_{a}\eta L}{A\left(P^{2}-P_{a}^{2}\right)}\left[m^{2}\right]$$

where

Q = V/t—the measured gas flow intensity [m^3^/s];

P_a_—atmospheric pressure [1 bar = 10^5^ Pa];

P—pressure (absolute) [Pa];

A—specimen cross-section area [m^2^];

η—viscosity of the gas; η = 17.15 [Pa·s];

L—specimen thickness [m].

**Figure 1:**
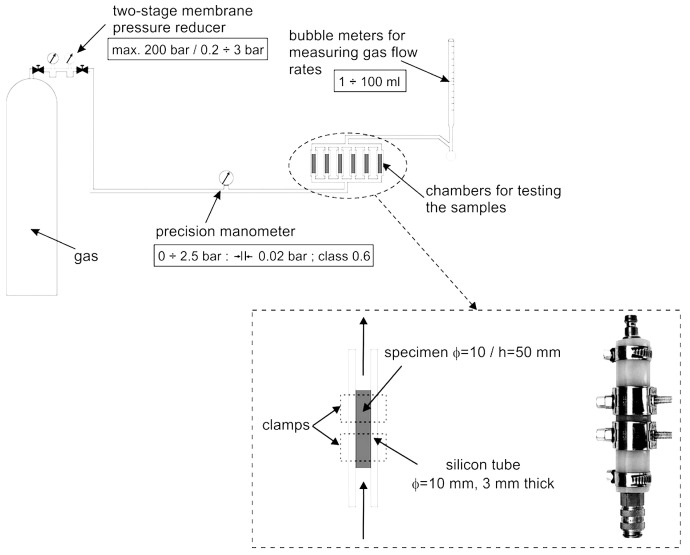
Equipment used for gas permeability testing based on RILEM-Cembureau method, along with specifics of securing specimens within a silicone tube chamber [46].

A schematic of the modified and adapted apparatus for measuring the permeability of 10 mm diameter specimens is shown in Figure 1. Except for the dimensions of the specimens, the testing protocol followed the guidelines outlined in the RILEM-Cembureau method, which is thoroughly detailed in [44].

For each sample, permeability measurements were performed separately. First, the gas was supplied from a single source. Then, the inlet gas pressure for each sample was kept constant, regardless of the permeability of individual samples. The measurement details are illustrated in the magnified view in Figure 1.

#### 2.2.2. Open Porosity

Open porosity was tested by three methods. The first method used a helium pycnometer, where porosity (pH) was calculated by comparing bulk (envelope) density with true (skeletal) density. In the second case, the mercury intrusion porosimetry method (pMIP) was used, and in the third case, open porosity was determined based on water saturation (pWS).

In the first stage, the saturation of the samples was carried out in a vacuum chamber, where the samples were subjected to vacuum conditions for 3 h at a reduced pressure of 20 mbar. Then, the samples were completely immersed in water, and after approximately 30 min, atmospheric pressure was restored, continuing the saturation process until a constant mass was achieved.

The helium porosity (pH) was determined based on the following formula:

(7) $$p_{H}=\left(1-\frac{\rho_{bulk}}{\rho_{true}}\right)100\left[\%vol.\right]$$

where

ρ_bulk_—bulk density [g/cm^3^],

ρ_true_—true density (helium pycnometry) [g/cm^3^].

Bulk density was determined using the Micromeritics GeoPyc 1360 powder pycnometer (Norcross, Georgia) [45], and true density was determined using the Quantachrome Ultrapycnometer 1200e helium pycnometer(Boynton Beach, FL, USA). This device allows a measurement of skeletal volume of the material to be conducted by filling open pores as small as 0.25 nm in diameter with helium molecules [11].

Mercury intrusion porosimetry (MIP) provides a wealth of relevant information on the porosity distribution of materials over a wide range from 3.75 nm to about 0.25 mm. The Quantachrome Poremaster 60 mercury porosimeter(Boynton Beach, FL, USA) with a pressure range from 0.1 to 400 N/mm^2^ was used in the tests.

#### 2.2.3. Microstructural Testing

Thermal analysis (TG-DTA) was conducted with the NETZSCH STA 449 F3 Jupiterinstrument (Selb, Germany), integrated with the QMS 403 Aëolos mass spectrometer (Selb, Germany). In addition to thermogravimetric analysis, this kit also allows analysis of evolved gases. The experiments were conducted across a temperature span from ambient temperature to 1000 °C, with a heating rate of 15 °C per minute. The resulting data enabled identification of the temperature intervals corresponding to successive stages: dehydration (Ldh) of, inter alia, ettringite, monosulphate, hydrogranates and the C-S-H phase, dehydroxylation (Ldx), primarily of portlandite, and decarbonation (Ldc) of calcite which appears in the paste as a result of the carbonation of mainly portlandite, but also of the C-S-H phase, hydrogranates or ettringite.

**Figure 2:**
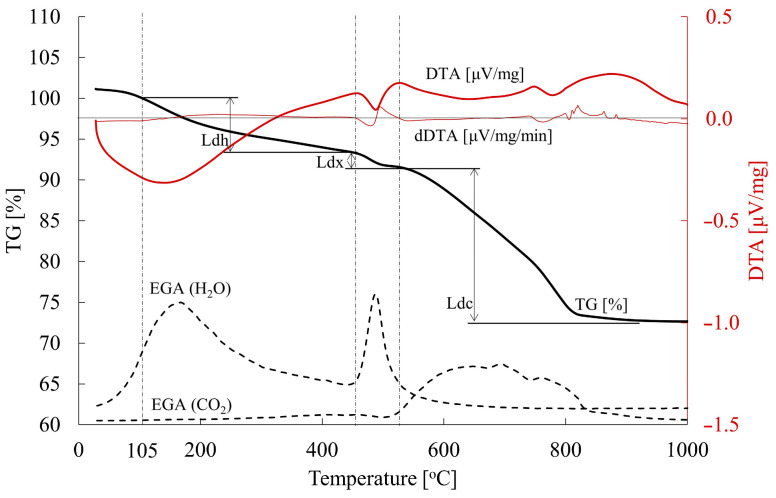
TG/DTA and EGA curves vs. temperature and their quantitative interpretation [40].

To accurately identify the temperature intervals corresponding to Ldx dehydroxylation, followed by the Ldh and Ldc phases, the derivative of the DTA curve was computed for each case. This allowed the use of zero dDTA values, along with the EGA profiles of H_2_O and CO_2_ released during heating, to pinpoint the onset and completion of portlandite decomposition (see Figure 2). Prior to thermogravimetric analysis, the samples were dried to constant mass in a vacuum laboratory oven at a temperature of 40 °C and a pressure of 20 mbar. Based on the problem of free water removal widely discussed in [46], a temperature of 105 °C was taken as the start of the dehydration process. To eliminate adsorbed moisture introduced during grinding, the measurement was initiated at a temperature of 30 °C. The mass loss observed up to 105 °C averaged approximately 1% and was excluded from subsequent calculations of the Ldh value.

On the basis of Equations (8) and (9) adopted by [47,48], the proportions of portlandite and products of the carbonation process, i.e., carbonates expressed as CaCO_3_, were calculated.

(8) $$CH\left[\%\right]=\frac{M_{Ca\left(OH\right)2}}{M_{H2O}}\cdot Ldx=4.11\cdot Ldx$$

where

*Ldx*—dehydroxylation mass loss [%]

*M_Ca(OH)_*_2_—calcium hydroxide molar mass (75.09 g/mol)

*M_H_*_2*O*_—water molar mass (18.02 g/mol)

(9) $$CaCO_{3}\left[\%\right]=\frac{M_{CaCO3}}{M_{CO2}}\cdot Ldc=2.27\cdot Ldc$$

where

*Ldc*—decarbonation mass loss [%]

*M_CaCO3_*—calcium carbonate molar mass (100.09 g/mol)

*M_CO2_*—carbon dioxide molar mass (44.01 g/mol)

The extent of cement hydration (α) was assessed based on Bhatty’s [49] approach, which involves computing the quantity of chemically bound water (WB) as defined by Equation (10).

(10) $$\alpha =\frac{W_{B}}{W_{B\infty }}\cdot 100$$

(11) $$W_{B}=Ldh+Ldx+0.41\cdot Ldc$$

where

*W_B_*—chemically bound water at time t [%]

*W_B∞_*—chemically bound water after completed hydration [%]

According to literature reports, the amount of chemically bound water after the hydration of OPCs and blended cements has been completed is variable, ranging from 0.18 to 0.23 [46,50,51,52,53], which is influenced, among other things, by the temperature at which these processes occur. On the basis of the aforementioned research, for the binders labelled as CEM I, CEM II, and CEM III, *W_B∞_* values of 0.22, 0.19, and 0.26, respectively, were adopted. The value of the coefficient in Equation (11), which is 0.41, follows from the molar mass of thermally removed CO_2_ relative to the molar mass of H_2_O (crystallisation water) in portlandite.

The phase composition of the base materials was investigated using a PANalytical Aeris X-ray diffractometer (Malvern PANalytical, Lelyweg 1, Almelo, The Netherlands), equipped with HighScore Plus software (version 4.8, Malvern PANalytical B.V., Almelo, The Netherlands). The analysis utilized the PDF-4+ database provided by the International Centre for Diffraction Data (ICDD), as well as the open COD-Inorg 2025 database. Measurements were conducted over a 2θ range of 10–100°, with a step size of 0.003° and a counting time of 340 s per step, using Cu Kα radiation. After a curing period of either 90 days or 2 years, the cement paste samples intended for phase composition analysis were dried at 60 °C and subsequently ground in an agate mortar to a particle size of approximately 100 µm. For XRD analysis, the fraction below 20 µm was then separated by sieving.

## 3. Porosity, Permeability Study Results and Discussion

Table 4 summarises the results of measurements of true (skeletal) and bulk (envelope) densities, as well as the total (helium) porosity calculated from Equation (7). These results were compared with porosity determined by mercury intrusion porosimetry, as well as by water saturation. The Table also includes the results of gas permeability tests. All the results presented are the average value of three measurements and refer to tests conducted both after 90 days and after 2 years of curing.

**Figure 3:**
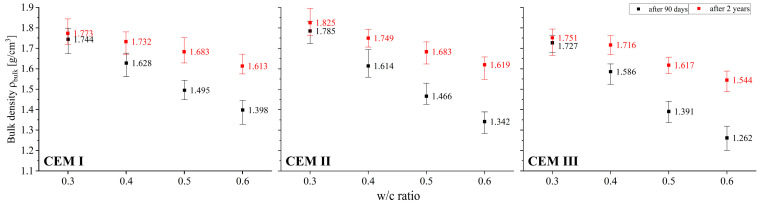
Relationship between bulk density and w/c ratio of cement pastes after 90 days and 2 years of curing.

**Figure 4:**
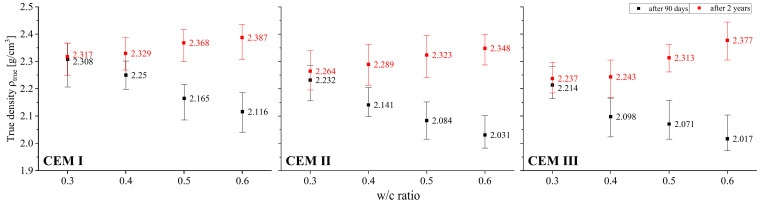
Relationship between true density and w/c ratio of cement pastes after 90 days and 2 years of curing.

**Figure 5:**
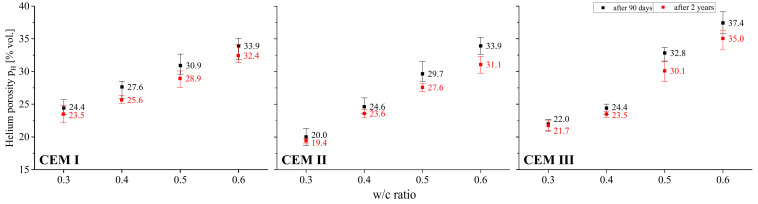
Relationship between helium porosity and w/c ratio of cement pastes after 90 days and 2 years of curing.

**Figure 6:**
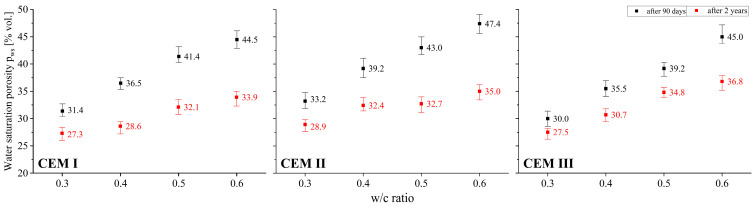
Relationship between water saturation porosity and w/c ratio of cement pastes after 90 days and 2 years of curing.

The obtained test results were characterized by high homogeneity adequate to the studied feature. Each time a set of values consisting of three measurements was verified in this regard. If there were results in the set that deviated from the average value by more than 10%, they were discarded. Then, after taking an additional measurement, an analogous homogeneity analysis was verified again. These steps were repeated until the criterion of ±10% relative to the mean value was met. While repeated measurements were not necessary for most of the features, in the case of the coefficient of permeability in several sets such a necessity to reject the results and repeat the tests occurred. As the study showed, this characteristic is extremely sensitive to changes in the internal structure, and therefore also to changes in the open porosity characteristics of the tested materials. To graphically present the uniformity of the obtained test results, all charts related to the density and porosity of the tested pastes (Figure 3, Figure 4, Figure 5 and Figure 6) include not only the average, but also minimum and maximum values.

### 3.1. Bulk Density

The test results shown in Figure 3. confirm that the w/c ratio has a very significant effect on the bulk density value irrespective of the paste curing time. The decrease in density caused by an increase in the w/c ratio is significant because when densities of pastes made using extreme w/c i.e., 0.3 and 0.6 values are compared, regardless of the type of cement used, it amounts to around 20% after 90 days of curing; after a longer period, this decrease is smaller and amounts to around 10%. The variation in bulk density observed for different curing periods appears quite interesting. For two-year curing, a higher level of cement hydration can be expected, but at the same time carbonation progresses as well, which was quantitatively confirmed in further studies. These simultaneous processes result in, inter alia, the increase in the bulk density of cement pastes after 2 years compared to the bulk density of pastes after 90 days. It should be noted that differences in these values rise together with the w/c ratio. In the case of pastes made from CEM I, bulk density after 2 years increased by 2% at a w/c ratio of 0.3, while for a paste with a w/c ratio of 0.6 this increase exceeded 15%. On the other hand, in the case of pastes made with blended cements, the increase was very similar at w/c = 0.3, while for the maximum amount of mixing water it was as high as 21% and 22% for CEM II and CEM III, respectively.

### 3.2. True Density

Since true density is a characteristic independent of material porosity, its value will be influenced by the densities of cement hydration products, carbonation of the individual ingredients, i.e., primarily portlandite and the C-S-H phase, and, especially in earlier curing phases, the density of relic cement grains. As can be seen in Figure 4, after 90 days of curing, true density for both pastes clearly decreases with increasing w/c. This trend is easily explained because when the amount of mixing water is increased, the amount of hydration products characterised by lower true density compared to non-hydrated cement grains also increases. Increasing hydration levels were confirmed by further thermogravimetric analyses. The opposite trend was observed after a 2-year curing period for pastes exposed to the natural progression of carbonation. The reason for the increase in true density with increasing w/c ratio is the crystallisation of carbonate salts. The migration of CO_2_ within pastes became easier as the w/c value increased, aided by the greater capillary porosity of the material.

The true densities of pastes made from blended CEM II and CEM III cements are very similar and exhibit similar qualitative and quantitative changes as a result of changes in their composition, i.e., w/c values, and as a result of further long-term progress of hydration and carbonation. This means that the observed density value is influenced less by relic binder grains with different initial densities, and more by the products of their hydration and crystallising carbonate salts. The true density of the pastes made from CEM I cement is somewhat higher than of those made from CEM II cement, and especially compared to the pastes made from CEM III cement. After 90 days of curing, CEM III cement pastes, essentially regardless of the w/c ratio, exhibit a density that is on average 5% lower than the density of CEM I cement pastes. Similar, though slightly smaller, differences were observed after 2 years of paste curing—they amounted to 2.5% on average. Similar relationships are observed when comparing pastes made from CEM I and CEM II cements where the average difference after 90 days is 4.0%, and after 2 years it is 1.9%.

### 3.3. Open Porosity

The following figures show the comparison between the open porosity of pastes determined by three methods, i.e., using helium and mercury, and finally saturating the specimens with water. The generally known phenomenon whereby open porosity increases together with an increase in the amount of mixing water, which at the same time implies a reduction in the amount of cement in the paste, was quantitatively confirmed. The observed changes in porosity determined by the helium method are clearly visible, with differences between pastes with a w/c of 0.3 and 0.6 exceeding 15%. In the case of CEM II cement pastes, open porosity after 90 days of curing was in the range from 20.0% to 33.9% by volume, while in CEM III cement pastes it was in the range from 22.0% to 37.4% by volume. The sensitivity of helium porosity to the value of the w/c ratio was the lowest in the case of CEM I cement pastes, with porosity ranging from 24.4% to 33.9%. After two years of curing, although the upward trend in helium porosity with increasing w/c continued, a decrease was noted irrespective of the w/c value and the type of cement tested. This reduction was greater the higher the value of the w/c ratio. This effect was most pronounced in pastes made with cements containing FA (fly ash) and GGBFS (ground granulated blast-furnace slag). For a w/c ratio of 0.3 the decrease in porosity for a paste made from CEM II cement after 2 years of curing amounted to 0.6% by volume, and for a w/c ratio of 0.6 it was 2.9% by volume. On the other hand, for a w/c ratio of 0.3 the decrease in porosity for a paste made from CEM III cement after 2 years of curing amounted to 0.3% by volume, and for a w/c ratio of 0.6 it was 2.4% by volume. The trends in changes in the porosity of cement pastes therefore depend on many factors, and a comprehensive quantitative assessment provides numerous interesting observations.

Like helium porosity, water saturation porosity is strongly influenced by the water-to-cement (w/c) ratio. An increase in this ratio leads to a higher amount of water uptake during paste absorption tests (see Figure 6). Surprisingly, water saturation porosity values compared to helium porosity of pastes with identical composition are higher. This is due to the fact that strongly polarised water molecules are adsorbed by the C-S-H gel, which results in the inter-packet spaces being filled, increasing their mutual distances. This results in a de facto increase in material porosity [44]. This effect was also confirmed in [18], where the swelling of cement binders stored in water was described.

The two-year curing period, which resulted in further progress in cement hydration and carbonation, contributed to a large reduction in water saturation porosity. These processes caused a drop in the porosity assessed in this manner by as much as 12.4 percentage points by volume in the case of CEM II cement paste with a w/c ratio of 0.6. In the case of CEM III paste with the same water to cement ratio, the reduction was the smallest at just 8.2% by volume. The reference binder showed a decrease of 10.6% by volume. In general, it can be observed that the degree to which this porosity is reduced depends not only on the type of cement, but also on the w/c ratio (see Figure 6), which in turn affects the potential for paste carbonation. After two years of curing, water saturation porosity is still higher than helium porosity, but the difference drops significantly. In general, the average difference between water saturation porosity and helium porosity is 10.4% by volume and ranges from 6.4% to 14.6% by volume after 90 days. In contrast, after 2 years, the variation averages 4.9% by volume and ranges from 1.5% to 9.5% by volume. The described effect may be due to the fact that long-term cement hydration, progressing simultaneously with carbonation, causes a reduction in the amount of C-S-H gel, which is susceptible to the increase in inter-packet spaces when filled with water.

**Figure 7:**
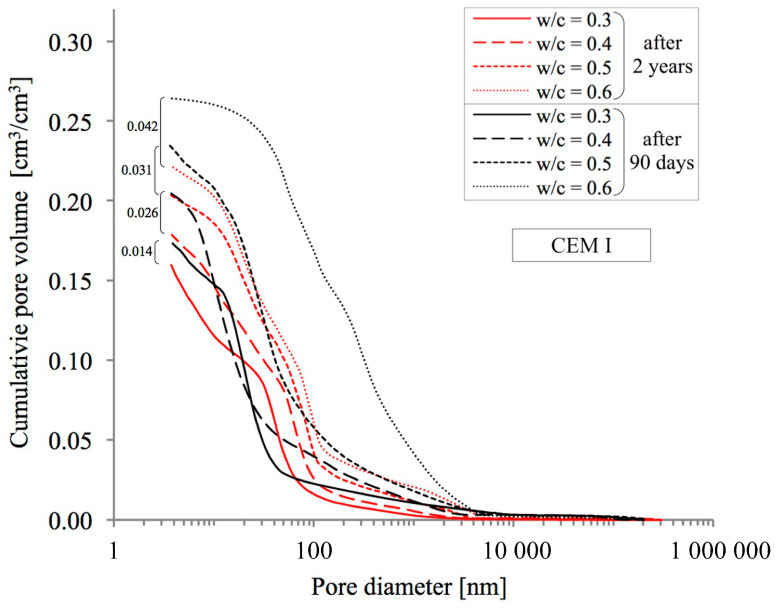
Pore distribution in cement pastes with w/c ratios of 0.3–0.6 made from CEM I cement after 90 days and 2 years of curing.

**Figure 8:**
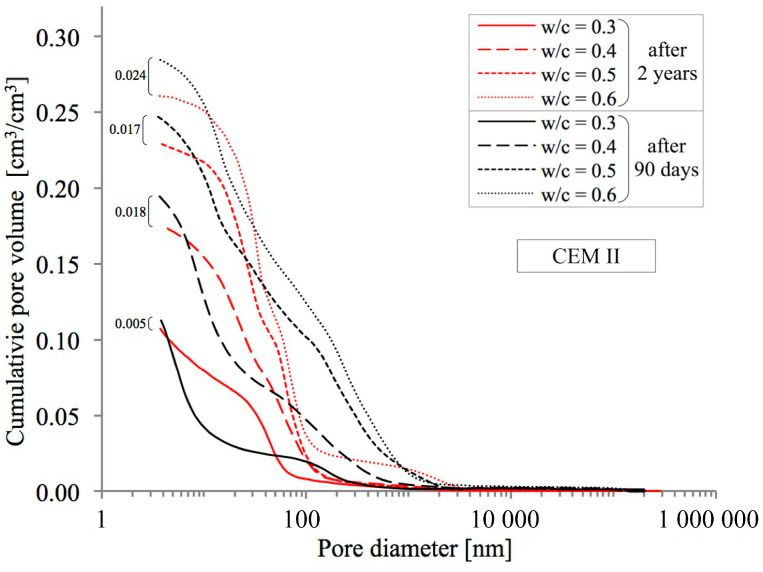
Pore distribution in cement pastes with w/c ratios of 0.3–0.6 made from CEM II cement after 90 days and 2 years of curing.

**Figure 9:**
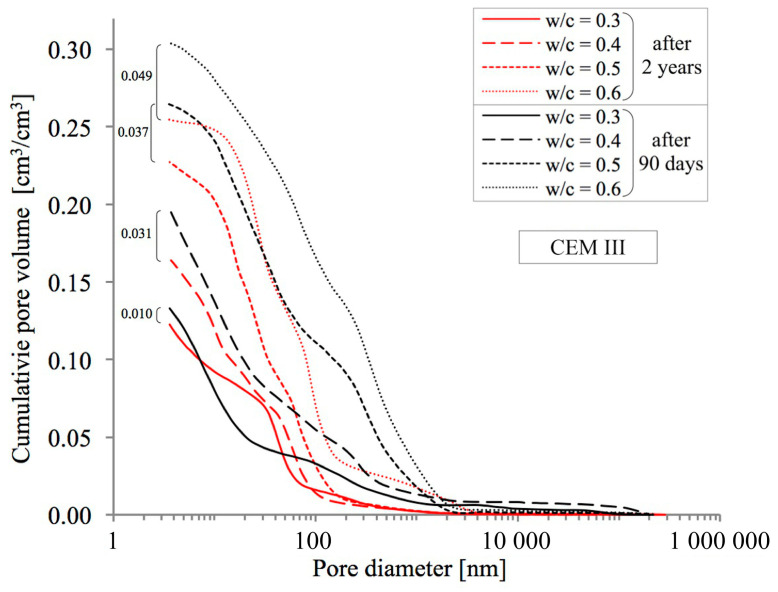
Pore distribution in cement pastes with w/c ratios of 0.3–0.6 made from CEM III cement after 90 days and 2 years of curing.

In order to obtain a full picture of the influence of the analysed phenomena of progressing hydration and simultaneous natural carbonation on the porosity of pastes, an MIP analysis was conducted, allowing changes in the distribution of pore diameters in the range from 3.75 nm to about 0.25 mm to be assessed quantitatively. Cumulative pore distribution curves are presented in Figure 7, Figure 8 and Figure 9 below.

As in the case of the above-described methods for testing total porosity, the observed upward trend in porosity with increasing w/c ratio values was confirmed as well. As can be seen in Figure 7, Figure 8 and Figure 9, after two years of hydration progress and parallel carbonation, total porosity decreases, which has also been noted by many other researchers [54,55]. The decrease in porosity is the greater the higher is the w/c ratio. For example, in a CEM III cement paste with a w/c ratio of 0.6, the decrease is 4.9 percentage points (from 30.4% to 25.5% by volume), which is similar to the value for an identical paste made from reference CEM I cement, where the decrease amounts to 4.3 percentage points (from 26.5% to 22.2% by volume). The smallest impact of hydration and carbonation on total porosity was observed in pastes made from CEM II with a w/c ratio of 0.6, where a decrease of just 1.4 percentage points (from 28.4% to 27.0% by volume) was recorded.

**Figure 10:**
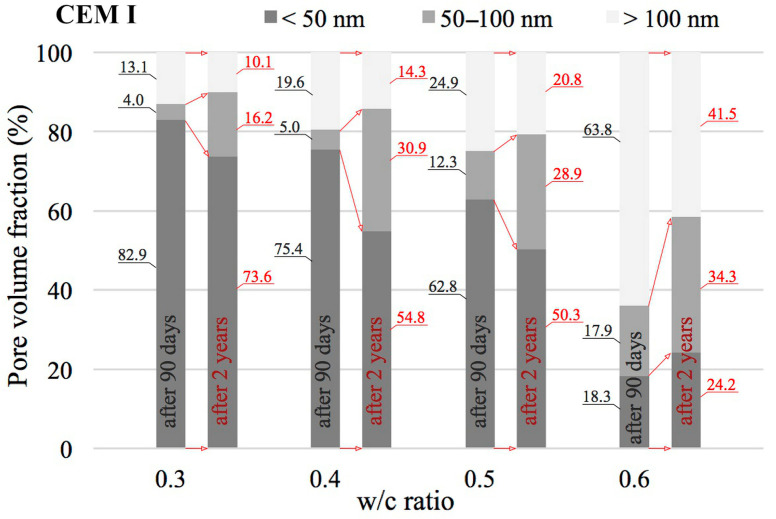
Distribution of pore types in CEM I cement pastes with differing water-to-cement ratios, evaluated after 90 days and 2 years of curing [40].

**Figure 11:**
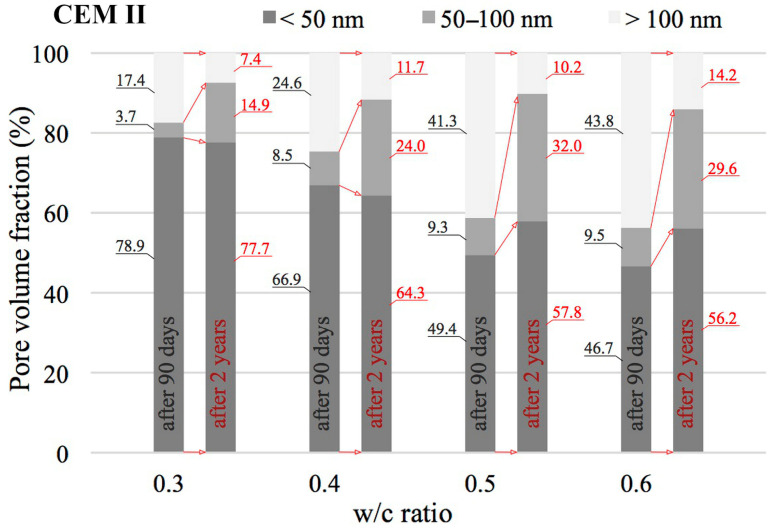
Distribution of pore types in CEM II cement pastes with differing water-to-cement ratios, evaluated after 90 days and 2 years of curing.

**Figure 12:**
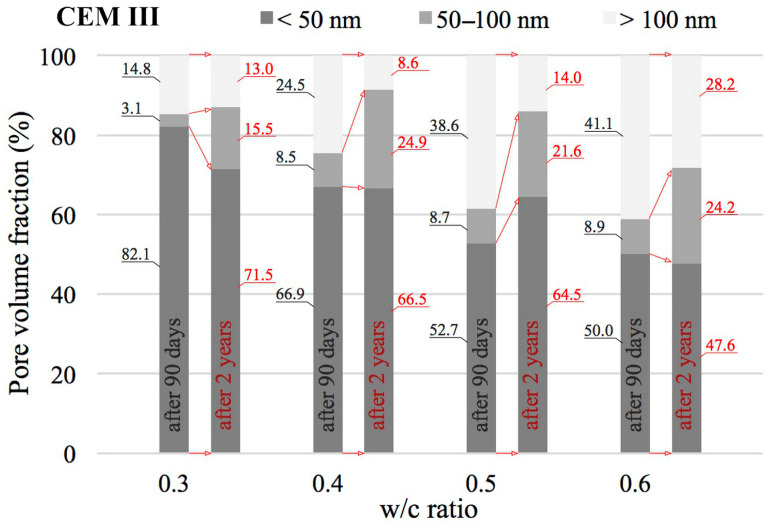
Distribution of pore types in CEM III cement pastes with differing water-to-cement ratios, evaluated after 90 days and 2 years of curing.

The value of the w/c ratio also significantly affects the change in porosity distribution as illustrated by cumulative curves. According to [24,56], the entire range of pores was divided into three classes: mesopores (<50 nm), middle capillary pores (50–100 nm) and larger capillary pores (>100 nm). The results of this analysis for pastes with different w/c ratios, made from different cements and after two different curing periods are shown in Figure 10, Figure 11 and Figure 12.

An increase in the w/c ratio results in an increase in the proportion of capillary porosity in all cement pastes analysed. Many researchers observe a similar trend [20,24,57]. After 90 days, in reference pastes with w/c = 0.3 capillary pores (>50 nm) accounted for only 17.1%, while an increase in w/c to 0.6 increased the proportion of capillary pores to 81.7%. In the case of pastes made with blended cement, no such sharp jump was observed after 90 days. In the case of pastes made from CEM II cement, the proportion of capillary pores for a w/c ratio of 0.3 is 21.1% and for a w/c ratio of 0.6, this proportion is 53.3%. On the other hand, in the case of pastes made from CEM III blast-furnace cement, the proportion of capillary pores for a w/c ratio of 0.3 is 17.9% and for a w/c ratio of 0.6, this proportion is 50.0%. From the above comparison, it is clear that the highest proportion of capillary pores is present in pastes made from CEM I cement. The addition of silicate fly ash, which is contained in CEM II/A-V cement (with a share of about 20%) significantly reduces the proportion of capillary pores compared to CEM I Portland cement pastes, but only at higher w/c ratios of 0.5 and 0.6. However, when proportions of capillary pores (>50 nm) in pastes made from Portland cement and from cement blended with GGBS are compared, a reduction of this proportion in favour of pastes made from CEM III cement is in fact only noticeable at a w/c ratio of 0.6. After 90 days, the proportion of medium capillary pores (50–100 nm) in the analysed cement pastes, relative to all capillary pores identified by the MIP method (i.e., >50 nm), is similar and ranges from 0.17 to 0.33. No trend was observed indicating that this proportion depends on the water-to-cement ratio (w/c). For the reference cement, this share averaged 0.25, while in pastes made with blended cements it was 0.20.

It was also observed, based on the pore size distribution curves presented in Figure 7, Figure 8 and Figure 9, that the threshold pore size decreases with a reduction in the water-to-cement ratio (w/c). This refers to the pore size at which significant filling of the open pore system with mercury begins in the tested material. Many authors use this parameter to describe the pore distribution in cement pastes [20,24,57]. Ren et al. [54] also observed a significant reduction in the threshold pore size in cement mortars subjected to accelerated carbonation.

Noteworthy shifts in the distribution of pore classes are evident when comparing the data collected at 90 days and after 2 years of curing. The data presented in Figure 10, Figure 11 and Figure 12 indicate that extending the curing period to two years led to a marked decrease in the share of large capillary pores exceeding 100 nm. In the case of pastes made from CEM I cement with a w/c ratio of 0.3, the decrease is from 13.1% to 10.1%, and for a w/c ratio of 0.6 the initial value of 63.8% drops to 41.5%. Similar changes can also be observed in pastes made from CEM II and CEM III cements. It can therefore be concluded that as open porosity increases, the changes in pore structure resulting from ongoing hydration and carbonation processes become more pronounced. The greatest decrease in the proportions of larger capillary pores was recorded for pastes containing FA with w/c ratios of 0.5 and 0.6, where initial values of 41.3% and 43.8%, respectively, were reduced to 10.2% and 14.2%, respectively. Differences in the proportion of mesopores (<50 nm) in the pastes are not large. At low w/c values, the number of these pores slightly decreases, while at high values it slightly increases. The exception was the CEM III cement paste with a w/c ratio of 0.6, in which the proportion decreased slightly from 50.0% to 47.6%.

The research conducted shows that all the factors analysed, i.e., paste composition, the type of cement used, and the progress of hydration as well as that of carbonation significantly affect the distribution of pores, and thus, as shown in the next section, the mass transport capacity of the binders tested.

### 3.4. Gas Permeability

**Figure 13:**
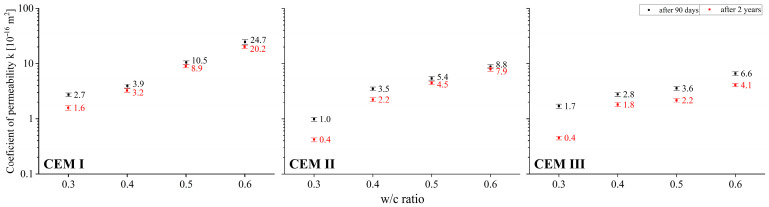
Relationship between permeability and w/c ratio of cement pastes after 90 days and 2 years of curing.

Since gas molecules can penetrate open pores as small as <1 nm [58], the RILEM-Cembureau method enables mass transport through cementitious composites to be analysed even for subtle changes in porosity distribution. Figure 13 presents the results of studies concerning the influence of material factors, i.e., the w/c ratio and type of cement, as well as hydration and carbonation time on the permeability of cement pastes.

Gas permeability—both after 90 days and two years of curing—strongly depends on the w/c ratio and on cement type. The relationship between the water-to-cement (w/c) ratio and the gas permeability of concrete was identified, among others, by Bakhshi et al. [59], and a strong correlation between these properties was found, qualitatively consistent with the results obtained from studies on cement pastes. Furthermore, experimental research conducted by Dutzer et al. [60] demonstrated that gas diffusion in cement pastes after carbonation is strongly dependent on the type of binder used. The greatest impact of the w/c ratio on permeability can be seen in the case of pastes made from CEM I Portland cement. Lower impact of the w/c ratio on permeability was recorded in the case of pastes made from CEM II and CEM III cements. Irrespective of the hydration time, at extreme w/c values, i.e., 0.3 and 0.6, the k coefficient increased around ten times for CEM I and CEM II cements, and around five times for CEM III. As it has been demonstrated above, a two-year hydration period combined with the simultaneous carbonation process cause significant changes in the porosity of the pastes analysed. These changes affect not only the total porosity, but also the distribution of pores, with a reduction in larger ones accompanied by an increase in mainly middle capillary pores observed. Permeability tests carried out after 2 years of curing show values lower than those recorded after 90 days, which is consistent with the observed changes in porosity distribution determined by the MIP method. The average decrease in permeability after 2 years compared to permeability after 90 days is around 34%, but it ranges considerably: from 11% to as high as 74%. The smallest differences in the permeabilities compared were recorded in the CEM II cement paste with a w/c ratio of 0.6, while the greatest were observed in the CEM III cement paste with a w/c ratio of 0.3. It should also be observed that in general, the greatest drops in permeability were recorded for pastes with a low w/c ratio, i.e., 0.3, irrespective of the type of cement used. Since the changes in total porosity and pore distribution in these pastes are very subtle, it is reasonable to believe that smaller pores in the sub-50 nm range will also play a role in the transport of gas molecules. According to [61], CaCO_3_ accumulates within the pore structure and leads to the closure of certain pores and cracks, particularly those with small diameters, which undoubtedly contributes to the reduction of gas permeability.

## 4. Results of Hydration and Carbonation Level Tests

### 4.1. XRD Test Results

Cement pastes made from CEM I, CEM II, and CEM III cements, which were characterised by extreme w/c ratio values, i.e., 0.3 and 0.6, were subjected to X-ray tests. The tests were carried out for paste specimens both after 90 days and after 2 years of curing.

**Figure 14:**
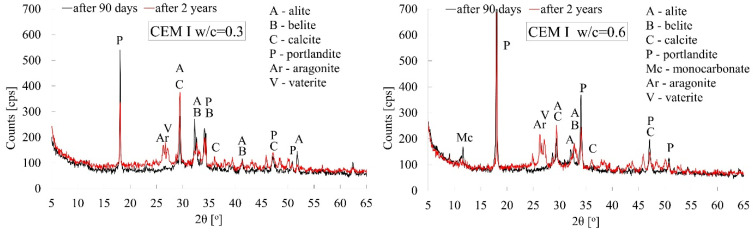
X-ray patterns of cement pastes made from CEM I cement with w/c ratios of 0.3 and 0.6 after 90 days and 2 years of curing [40].

**Figure 15:**
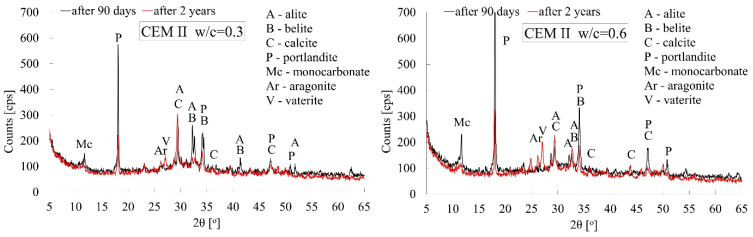
X-ray patterns of cement pastes made from CEM II/A-V cement with w/c ratios of 0.3 and 0.6 after 90 days and 2 years of curing.

**Figure 16:**
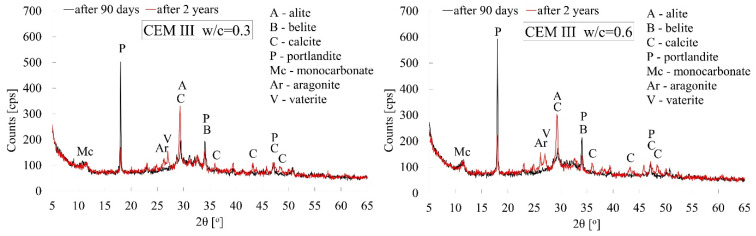
X-ray patterns of cement pastes made from CEM III/A cement with w/c ratios of 0.3 and 0.6 after 90 days and 2 years of curing.

The X-ray patterns shown in Figure 14, Figure 15 and Figure 16 below for all types of cements clearly indicate the progress of the carbonation process between 90 days and 2 years of curing under laboratory conditions. Irrespective of the type of cement used and the water–cement ratio, a significantly lower intensity of the peak associated with Ca(OH)_2_ portlandite is observed in favour of CaCO_3_ calcite (ICDD 00-047-1743). Thus, it can be concluded that the progress in carbonation between 90 days and two years of curing is dominant compared to the slow, diffusion-driven progression of the reaction between water molecules and the still-unhydrated relics of cement grains. The naturally increased porosity of pastes with high w/c ratio values, which boosts the kinetics of the carbonation process, is also reflected in the reduction of the intensity of the main peak which is characteristic of portlandite (2θ = 18°) (ICDD 00-002-0967) and the increase in the intensity of the peak attributable to calcite (2θ = 29°). In the case of cement paste made from CEM I, the reduction in the intensity of the portlandite peak was 40% greater when the w/c ratio was 0.6 than when it was 0.3. A similar situation occurred in the case of cement pastes made from CEM II and CEM III cements, with reductions of 20% and 10%, respectively. The progress of the carbonation process over the longer, 2-year curing period is also confirmed by the appearance of additional peaks characteristic of calcite in virtually all cases of pastes analysed.

In connection with the indications appearing in the literature regarding the occurrence of various polymorphic forms of calcium carbonate [62,63], in addition to the previously mentioned calcite, vaterite (COD 9017408) and aragonite (ICDD 00-001-0628) has also been observed after a longer period of carbonation time, i.e., after 2 years. All these polymorphic varieties of calcium carbonate were observed irrespective of the amount of mixing water used. It is also worth noting that at high w/c values, after 90 days of hydration, an unstable monocarbonate (COD 1000459) C_3_A·CaCO_3_·11H_2_O phase could be observed in all types of cement, which could have crystallised as a result of carbonation of hydrated calcium aluminates [31] and sulphoaluminate phases [64]. As indicated by X-ray tests, this phase tends to disappear over a longer period of time.

In addition, X-ray patterns also show a clear difference in the degree of hydration of cement pastes after 90 days of curing, which is determined by the amount of mixing water. In pastes made from CEM I or CEM II cements, the intensity of the peak attributable to portlandite is more than twice as high in the case of pastes with a w/c ratio of 0.6 than in those with a w/c ratio of 0.3, which indicates the degree of crystallisation of this cement hydration product. Cement pastes made from CEM II and CEM III cements exhibit a similar effect, but to a lesser extent. Additional confirmation is the disappearance of the characteristic peak attributable to alite (2θ = 51°). In the case of CEM I and CEM II cements, this is only visible after 90 days of hydration at w/c = 0.3. Both the two-year hydration time and the increased amount of mixing water, i.e., w/c = 0.6, cause this peak to disappear.

### 4.2. TG/DTA Test Results

**Figure 17:**
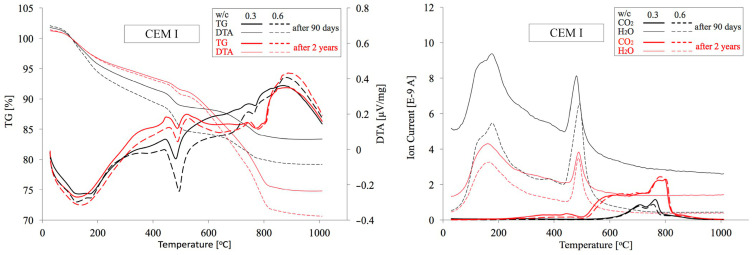
TG/DTA and EGA analyses of pastes made from CEM I cement with w/c ratios of 0.3 and 0.6 after 90 days and 2 years of curing.

**Figure 18:**
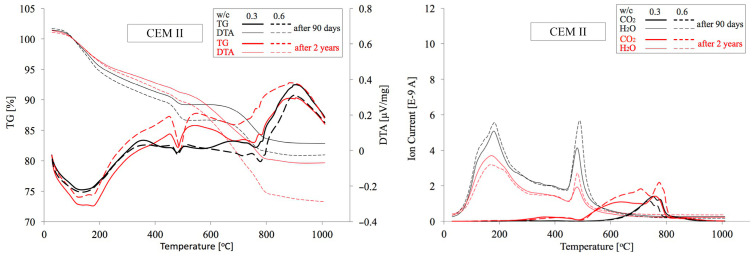
TG/DTA and EGA analyses of pastes made from CEM II cement with w/c ratios of 0.3 and 0.6 after 90 days and 2 years of curing.

**Figure 19:**
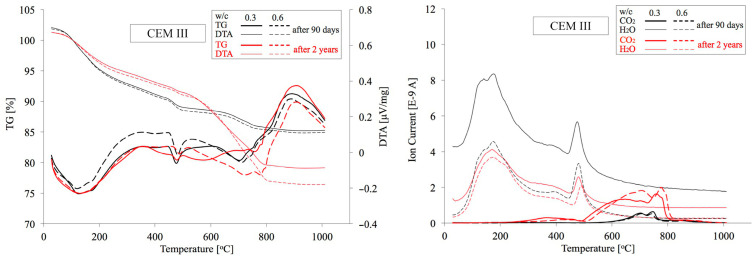
TG/DTA and EGA analyses of pastes made from CEM III cement with w/c ratios of 0.3 and 0.6 after 90 days and 2 years of curing.

Similarly, as in the previous case, analogous cement paste specimens made from CEM I, CEM II and CEM III cements with minimum and maximum w/c ratios of 0.3 and 0.6 were subject to thermogravimetric analysis. In Figure 17, Figure 18 and Figure 19 below, TG/DTA and EGA analyses of cement pastes are summarised. Table 5 presents their dehydration (Ldh), dehydroxylation (Ldx), and decarbonation (Ldc) levels within the temperature ranges determined in accordance with the procedure described in Section 2.2.3. On this basis, the quantities of portlandite and carbonates expressed as CaCO_3_, as well as the amount of chemically bound water and the degree of cement hydration α were calculated. Among the various available methods for preparing samples for microstructural analysis [65], the samples were dried to constant mass in a laboratory oven at 40 °C and 20 mbar. To eliminate moisture absorbed during grinding, the measurement was initiated at 30 °C. The mass loss up to 105 °C averaged only about 1% and was excluded from subsequent calculations of the Ldh value. The degree of carbonation of OPC, as well as the loss on ignition of FA and GGBFS, were also omitted from the calculations. These differences are negligibly small and affect the estimated content of both Ca(OH)_2_ and carbonates only at the second decimal place.

**Figure 20:**
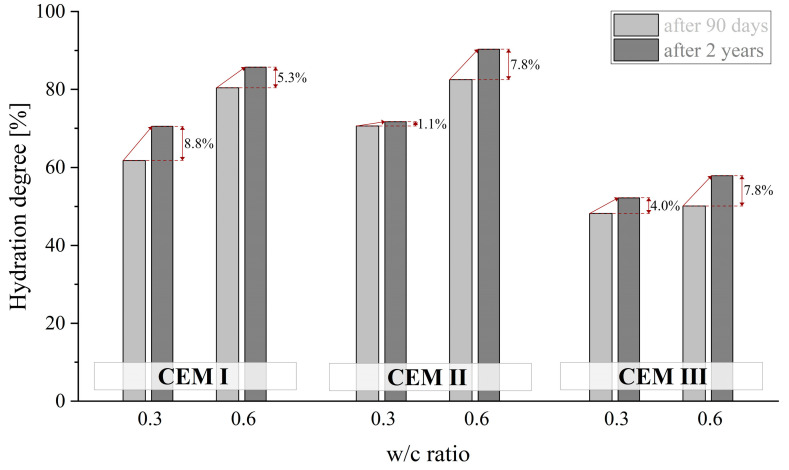
Degree of hydration of CEM I, CEM II and CEM III cements depending on w/c ratio after 90 days and 2 years of curing.

Irrespective of the type of cement used, a significant impact of both the curing time and the amount of mixing water on the degree of cement hydration α was observed (see Figure 20). The highest degree of hydration was exhibited by CEM II cement, reaching a value of more than 90% after 2 years of curing for a w/c ratio of 0.6. On the other hand, the lowest degree of hydration, i.e., around 60% for the same curing period and amount of mixing water used, was exhibited by CEM III cement, which, as is generally known, has a lower reaction kinetics with water. The value of the w/c ratio had the most impact on the hydration process in the case of the CEM I binder—after 90 days, an increase in α of 20 percentage points was recorded. The pastes made with CEM II and CEM III showed changes of around 12 percentage points and of just 2 percentage points, respectively. It is also worth noting that at w/c = 0.3, blended pastes showed a negligible increase in the degree of hydration during the 2-year observation period.

**Figure 21:**
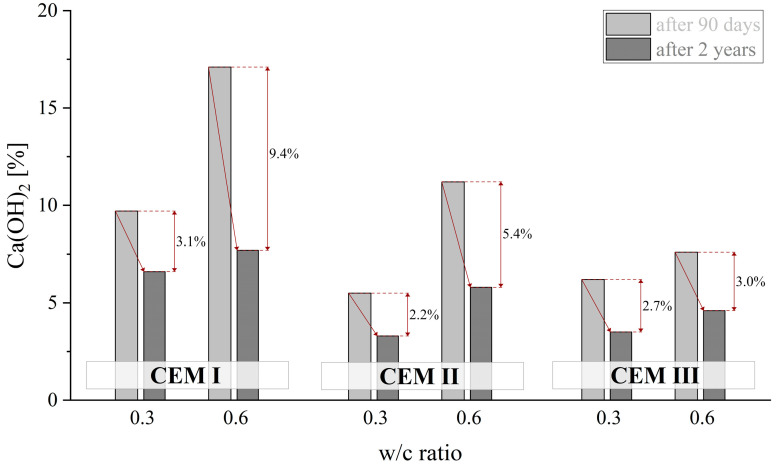
Content Ca(OH)_2_ of CEM I, CEM II and CEM III cements depending on w/c ratio after 90 days and 2 years of curing.

As can be observed in Figure 21, cement pastes containing SCMs exhibited a reduction of around 40% in the amount of CH caused by the progress of carbonation between 90 days and 2 years irrespective of the w/c ratio. The exception was CEM I cement where in the case of the w/c ratio of 0.3, this reduction was 30%, while it increased to more than 50% when more mixing water was used. The quantity of portlandite formed after 90 days of curing was also influenced by the volume of water used during mixing. When comparing pastes with w/c ratios of 0.3 and 0.6, this amount increases by approximately 100% in the case of pastes made from CEM I and CEM II cements, while for CEM III cement pastes it only increases by 20%. It should also be noted that the amount of crystallised CH for all analysed w/c ratio values, irrespective of curing time, was the highest for cement paste made from CEM I cement. Pozzolanic additives in CEM II cement reduced the amount of this phase by 25% to 50% depending on the curing time. In the case of paste made from CEM III cement, the limited amount of clinker and the relatively low hydration level resulted in CH precipitation that was between 40% and 55% lower than in the case of CEM I.

**Figure 22:**
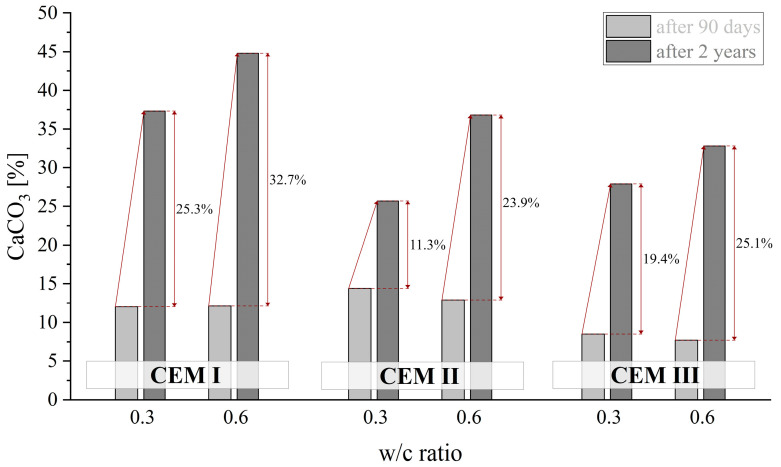
Content CaCO_3_ of CEM I, CEM II and CEM III cements depending on w/c ratio after 90 days and 2 years of curing.

Not surprisingly, irrespective of the type of cement used, for the highest water/binder values the most intense progression of carbonation was observed with a high w/c ratio of 0.6 (see Figure 22). This is mentioned, inter alia, by the authors of [66]. After two years of curing, the amount of carbonates increased 3.7 times for CEM I cement, whereas in pastes made from CEM II and CEM III cements, this value increased 2.8 and 4.2 times, respectively. The highest degree of carbonation (amount of carbonates in the paste) is exhibited by pastes made from CEM I cement, which is also mentioned by the authors of [67]. For a w/c ratio of 0.6, the amount of equivalent CaCO_3_ was as high as 45%. In these conditions, which are the most conducive to carbonation, the pastes made from CEM II and CEM III cements contained 37% and 33% of carbonates, respectively. According to the authors of [68], carbonation potential is reduced not only by the reduction of the w/c ratio, but also by the content of SCMs such as FA or GGBFS, which, having pozzolanic and/or latent hydraulic properties, are involved in the formation of an additional amount of the C-S-H or C-(A)-S-H phase, thereby sealing the structure of the material.

## 5. Discussion of Study Results

On the basis of literature reports [27,69,70,71] on the skeletal densities of the individual phases appearing during the carbonation process, which occurs according to Equations (1)–(5) as presented in the Introduction, a qualitative analysis of changes in the porosity of the cement pastes studied was carried out. Table 6 presents the molar masses, true densities and molar volumes of the phases involved in the aforementioned mechanisms underlying the carbonation process. On this basis, comparisons were conducted concerning the change in the volume of the products relative to the substrates in the solid state, i.e., excluding gaseous and liquid reactants (CO_2_ and H_2_O), which play a negligible role in the change in the porosity of pastes subjected to drying under the conditions described in Section 2. Since, as demonstrated by XRD studies, portlandite carbonation can result in the formation of three different polymorphic varieties of calcium carbonate in the paste structure, volume change calculations were performed for calcite, aragonite and vaterite.

Authors of individual publications are in agreement as concerns the qualitative phase composition of the hydrated cement paste produced from OPC, albeit with some quantitative variations: the content of the C-S-H phase oscillates around 30–40% by volume and that of CH ranges from 15% to 20% by volume, while the proportion of the monosulphate phase formed as a result of ettringite disappearing is around a dozen percent [31,72]. Thus, estimating the change in the volume of paste made from CEM I and subjected to carbonation, and assuming that calcite is the most commonly formed polymorphic variety of calcium carbonate, it can be claimed that a global increase in the volume of the products occurs relative to the substrates. This increase, taking into account the volume shares of the individual phases, amounts to around 8%. Further carbonation progression could have the opposite effect. According to [73], in the presence of CH and CaCO_3_, monosulphate can form ettringite crystals within the cement paste structure along with monocarbonate. This one, in turn, undergoes further decomposes into CaCO_3_ and aluminium hydroxide. In Reaction (4), 3 moles of monosulphate result in 1 mole of the AFt phase being formed, which, according to the data presented in Table 7, represents 75% of substrate volume. Even assuming a complete decomposition of AFm and its transformation to ettringite, followed by a further decomposition of this phase to calcium carbonate, aluminium hydroxide and gypsum as per Reaction (5), the global volume balance of all products relative to the substrates, taking into account their proportions in the hardened paste, remains positive. However, the increased content of aluminate phases in the presence of FA or GGBFS could tip the phase volume balance in favour of the substrates. It should also be mentioned that irrespective of its polymorphic variety, crystallising calcium carbonate, upon further access to H_2_O and CO_2_, can undergo further recrystallisation to calcium bicarbonate whose water solubility is two orders of magnitude higher. In the case of the pastes tested, no leaching process occurred due to the conditions under which the natural carbonation process progressed, i.e., under ambient conditions where the relative humidity ranged from 50 to 70%. The fact that total porosity of pastes subjected to carbonation is reduced irrespective of the type of binder used and the values of the w/c ratio confirms the changes described. Thus, it can be assumed that in the case of CEM II and CEM III cements, the proportion of hydrated calcium aluminates does not determine changes in paste porosity to such an extent as to lead to its increase, or the period during which natural, slow carbonation was studied was not long enough to bring about a sufficient decomposition of the AFm and (subsequently) AFt phases.

Changes in the porosity distribution of all pastes examined primarily relate to the reduction of mesopores in favour of middle capillary pores. As reported by the authors of [68], the reduction in micropores is mainly due to the formation of fine calcium carbonate crystals as a result of the decomposition of the C-S-H phase. In turn, the increase in the content of middle capillary pores can be attributed to the mechanism whereby microcracks appear that are initiated in the CaCO_3_ layer around CH as a result of hydrostatic pressure (hydraulic gradient) and shrinkage caused by carbonation (release of water molecules). A third phenomenon reported in the literature that can affect changes in porosity at the larger capillary pore level is the precipitation of relatively large calcium carbonate crystals as a result of portlandite decomposition [68].

Irrespective of the type of cement used—CEM I, CEM II, or CEM III—the appearance of hydration products entails a reduction in the true density of the products relative to the substrates (see Table 4 and Table 6). Thus, it is easy to explain the relationships presented in Figure 4 between the 90-day true density and the water/binder ratio. This is because the pronounced reduction shown is associated with an increase in the degree of hydration of the cements studied.

After two years of natural carbonation, however, this density increases for all types of binders, and the level of increase is strongly determined by the initial amount of mixing water, and thus the carbonation potential of the material examined. This potential was confirmed by thermogravimetric analyses (see Table 5), where, as shown, cement pastes made with CEM I, CEM II, and CEM III cements at w/c = 0.6 showed 20, 40, and 17% higher carbonate contents expressed as CaCO_3_, respectively, compared to pastes made with w/c = 0.3. The largest increase in the density of the carbonated skeleton of the hardened pastes was observed when 50% of OPC was replaced by GGBFS. The increase in the amount of SCMs that are Al carriers promotes the formation of greater amounts of hydrated calcium aluminate phases such as hydrogranates, monosulphate and ettringite, characterised by the (relatively) lowest true density ranging from 1.78 to 2.05 g/cm^3^. On the other hand, crystallisation of calcite, aragonite or vaterite is associated with an increase in true density to 2.54–2.93 g/cm^3^. In the case of C-S-H phase or portlandite decomposition, the change in true density is not as pronounced.

The progress of carbonation of the pastes analysed appeared to be a secondary factor affecting their gas permeability compared to the type of cement used and the value of the w/c ratio. Each time, a reduction in gas permeability was observed, which was related to the progress of paste hydration on the one hand, and to reaction with CO_2_ on the other. For both processes, V_prod_/V_sub_ > 1. The average reduction in permeability after two years of natural carbonation was about 34%, and ranged from 11% to 74%. The greatest drops in permeability were recorded for pastes with a low w/c ratio, i.e., 0.3, irrespective of the type of cement used. It was in these pastes made from CEM I, CEM II, and CEM III cements that permeability drops of 42%, 58%, and 74%, respectively, were recorded. The reason for the phenomenon observed may be the nucleation of calcium carbonate in mesopores, resulting primarily from the decomposition of the C-S-H phase. As the results obtained demonstrate, total porosity is not the only parameter that determines the permeability of cement pastes, which is also confirmed by the authors of [74]. For CEM III cement at w/c = 0.4, total porosity determined by the helium pycnometer method was less than 25% after 90 days, while at w/c = 0.5 the P_H_ value was more than 5 percentage points higher after 2 years. However, the relationship was reversed when comparing the permeability of the two materials. The results obtained confirm the opinion presented by the authors of [75,76] that mass transport capacity is a function not only of the total number of open pores, but also of their distribution.

## 6. Conclusions

One should bear in mind that the quantitative changes described relate to the binder of the cementitious composites in the bulk. In realistic aggregate-containing materials, it is usually the interfacial transition zone that is most porous and also characterised by the highest permeability. This can largely determine the durability of the entire composite. The limitations of the study also include the period and method of the carbonation process. A two-year period was chosen for the reaction of the pastes with CO_2_ under laboratory conditions. For this reason, the results obtained do not relate to fully carbonised materials. Moreover, the kinetics of this process is strongly dependent on both the RH of the surrounding environment and its variability, as well as the temperature. For this reason, a similar lifetime of cementitious composites under real conditions will not necessarily provide identical levels of hydration as well as carbonation as in the case of the model pastes tested.

The objective of the research and analysis carried out was to determine quantitative changes in permeability in cement pastes made from typical cements at varying w/c ratios, occurring as a result of a long natural carbonation process. At the same time, microstructural changes in the pastes analysed and their impact on mass transport capacity were described.

As concerns the gas permeability of pastes, the essential factor determining this characteristic is the composition of the cement, i.e., the presence of SCMs. Cement pastes made with CEM II and CEM III were much less susceptible to the amount of mixing water, which was the second most significant parameter affecting the change in permeability compared to CEM I. Irrespective of the type of cement used and the value of the w/c ratio, the two-year curing period of pastes exposed to natural effects of CO_2_ resulted in a reduction in transport capacity due to two processes, i.e., further progression of hydration, and the simultaneous carbonation. As demonstrated earlier, in the case of both reactions the volume ratio of products to substrates V_prod_/V_sub_ > 1, which explains the changes in permeability that occurred. The decrease of the k coefficient ranged from 11% to 74% depending on the type of cement and the w/c ratio. In addition, for lower w/c values, i.e., in tight pastes characterised by low open porosity and at the same time a high proportion of mesopores, the relative reduction in permeability was the most pronounced.

The results of the thermogravimetric analyses conducted confirmed that CEM I cement showed the highest CO_2_ binding capacity under natural conditions over a period of 2 years. For both extreme values of the w/c ratio (0.3 and 0.6), a carbonate content that was about 10 percentage points higher compared to the blended pastes was observed. Referring to the effect of the amount of mixing water on the CO_2_ binding capacity of the pastes examined, irrespective of the type of binder used, for a higher w/c value the amount of carbonates became naturally higher, but the difference did not exceed 11 percentage points for the extreme w/c values.

The CEM I paste with w/c = 0.6 showed the greatest carbonation potential not only because of its highest permeability, but also because of the portlandite content in the structure of the hardened paste. This phase exhibits the highest reactivity towards carbon dioxide, and its content was reduced by more than half after 2 years of exposure.

Carbonation of portlandite, the C-S-H phase, and monosulphate leads to a volumetric expansion of the reaction products compared to the original compounds, which not only reduces overall open porosity but more significantly alters the pore structure distribution. Carbonation causes a significant reduction in the proportion of larger pores in favour of middle capillary pores. These changes are greater the more pores >100 nm are present in the paste structure, i.e., for high w/c values.

The carbonation of cement paste ingredients and the further progress of hydration of relict cement grains, resulting in a significant reduction in capillary pore amount, leads to a reduction in water absorption by the pastes analysed. The decrease in water saturation porosity as a result of these processes amounted to 18% on average, and the recorded variation increased together with the porosity exhibited by the paste after 90 days of curing. Still, the values of total open porosity determined in this manner are slightly higher than those of helium porosity in spite of the fact that water penetrates pores with much larger diameters than helium. After the longer, two-year period of curing, the difference related to these characteristics is significantly lower. This effect can be explained by the reduced susceptibility of the ingredients included in cement paste microstructure to the emergence of “additional porosity”, which arises as a result of liquid being absorbed by the hydrated calcium silicate gel, which results in filling the inter-packet spaces and thus increasing the distances between them.

## Figures and Tables

**Table 1:** Chemical characteristics of OPC, FA, and GGBFS [40].

Oxides	OPC [%]	FA [%]	GGBFS [%]
SiO_2_	18.6	51.9	41.6
Al_2_O_3_	5.3	31.9	8.6
Fe_2_O_3_	2.9	5.1	0.6
CaO	62.7	2.6	39.1
MgO	1.50	1.4	6.8
SO_3_	3.22	0.8	1.7
Na_2_O	0.19	1.1	0.6
K_2_O	0.96	2.3	0.4
Na_2_O_eq_	0.82	2.5	0.8
Cl^−^	0.06	0.02	0.01
LOI	2.8	3.6	0.3

**Table 2:** Physical properties of blended cements.

Oxides	CEM I	CEM II	CEM III
Additive content (FA, GGBFS)	-	20	50
Specific area (Blaine method), m^2^/kg	340	366	465
True density, g/cm^3^	3.09	2.97	2.97
Setting time (minutes)			
-start	199	176	221
-end	270	221	266
Compressive strength, N/mm^2^			
-after 2 days	29.3	25.4	13.7
-after 28 days	55.1	56.2	50.7

**Table 3:** Composition of cement pastes.

w/c Ratio	Cement [kg/m^3^]	Water [dm^3^/m^3^]
0.30	1605	483
0.40	1384	554
0.50	1217	608
0.60	1083	650

**Table 4:** Results of density, porosity, and permeability tests for cement pastes tested [40].

Cement Type	w/c Ratio	Bulk Density ρ_bulk_ [g/cm^3^]	True Density ρ_true_ [g/cm^3^]	Helium Porosity p_H_ [% vol.]	MIP Porosity p_MIP_ [% vol.]	Water Saturation Porosity p_WS_ [% vol.]	Coefficient of Permeability k [10^−16^ m^2^]
after 90 days							
CEM I	0.3	1.744	2.308	24.4	17.3	31.4	2.73
	0.4	1.628	2.250	27.6	20.5	36.5	3.95
	0.5	1.495	2.165	30.9	23.5	41.4	10.50
	0.6	1.398	2.116	33.9	26.5	44.5	24.70
CEM II	0.3	1.785	2.232	20.0	11.3	33.2	0.99
	0.4	1.614	2.141	24.6	19.5	39.2	3.50
	0.5	1.466	2.084	29.6	24.7	43.0	5.39
	0.6	1.342	2.031	33.9	28.4	47.4	8.83
CEM III	0.3	1.727	2.214	22.0	13.3	30.0	1.70
	0.4	1.586	2.098	24.4	19.5	35.5	2.79
	0.5	1.391	2.071	32.8	26.4	39.2	3.57
	0.6	1.262	2.017	37.4	30.4	45.0	6.63
after 2 years							
CEM I	0.3	1.773	2.317	23.5	16.0	27.3	1.58
	0.4	1.732	2.329	25.6	17.9	28.6	3.25
	0.5	1.683	2.368	28.9	20.4	32.1	8.93
	0.6	1.613	2.387	32.4	22.2	33.9	20.15
CEM II	0.3	1.825	2.264	19.4	10.7	28.9	0.42
	0.4	1.749	2.289	23.6	17.6	32.4	2.22
	0.5	1.683	2.323	27.6	23.0	32.7	4.45
	0.6	1.619	2.348	31.0	27.0	35.0	7.86
CEM III	0.3	1.751	2.237	21.7	12.3	27.5	0.45
	0.4	1.716	2.243	23.5	16.4	30.7	1.80
	0.5	1.617	2.313	30.1	22.7	34.8	2.15
	0.6	1.544	2.377	35.0	25.5	36.8	4.06

**Table 5:** Thermogravimetric analysis results.

Cement Type	w/c Ratio	Ldh [%]	Ldx [%]	Ldc [%]	Ca(OH)_2_ [%]	CaCO_3_ [%]	α [%]
after 90 days							
CEM I	0.3	9.04	2.37	5.29	9.7	12.0	61.7
	0.6	11.32	4.17	5.35	17.1	12.1	80.4
CEM II	0.3	9.48	1.33	6.35	5.5	14.4	70.6
	0.6	11.53	2.94	6.70	11.2	12.9	82.5
CEM III	0.3	9.49	1.50	3.74	6.2	8.5	48.2
	0.6	9.80	1.84	3.41	7.6	7.7	50.1
after 2 years							
CEM I	0.3	7.16	1.60	16.44	6.6	37.3	70.5
	0.6	6.74	1.70	18.93	7.7	44.8	85.7
CEM II	0.3	8.17	0.81	11.34	3.3	25.7	71.7
	0.6	7.48	1.16	14.60	5.8	36.8	90.3
CEM III	0.3	7.67	0.86	12.30	3.5	27.9	52.2
	0.6	8.03	1.11	14.45	4.6	32.8	57.9

**Table 6:** True densities and molar volumes of basic phases present in cement paste.

Phase	Composition Formula	Molar Mass [g/mol]	True Density [g/cm^3^]	Molar Volume [cm^3^/mol]
Portlandite	CH	74	2.23	33.2
C-S-H phase	C_1.7_SH_2.5_	200	2.41	83.0
Calcite	CaCO_3_	100	2.71	36.9
Aragonite	CaCO_3_	100	2.93	34.1
Vaterite	CaCO_3_	100	2.54	39.4
Amorphous silica	SiO_2_	60	2.20	27.3
Hydrogranate	C_4_AH_13_	560	2.05	273.2
Ettringite	C_3_A·3(C$\bar{S}$)·H_32_	1254	1.78	704.5
Monosulphate	C_3_A·C$\bar{S}$·H_12_	622	1.99	312.6
Aluminium hydroxide	Al(OH)_3_	78	2.42	32.2
Gypsum	C$\bar{S}$·H_2_	172	2.31	74.5

**Table 7:** Changes in volumes of substrates and solid products resulting from carbonation processes.

No.	Solid Substrates		Solid Products	V_prod(Cal/Ar/Vat)_/V_sub_
(1)	Ca(OH)_2_	→	CaCO_3 (Cal/Ar/Vat)_	1.11/1.03/1.19
	V_sub_ = 33.2		V_prod_ = 36.9/34.1/39.4	
(2)	1.7CaO·SiO_2_·2.5H_2_O	→	1.7CaCO_3 (Cal/Ar/Vat)_ + SiO_2 (am)_	1.08/1.03/1.14
	V_sub_ = 83.0		V_prod_ = 90.0/85.3/94.2	
(3)	4CaO·Al_2_O_3_·13H_2_O	→	4CaCO_3 (Cal/Ar/Vat)_ + 2Al(OH)_3_	0.78/0.74/0.81
	V_sub_ = 273.2		V_prod_ = 212.1/201.0/221.9	
(4)	3(3CaO·Al_2_O_3_·CaSO_4_·14H_2_O)	→	3CaO·Al_2_O_3_·3CaSO_4_·32H_2_O + 6CaCO_3 (Cal/Ar/Vat)_ + 2Al(OH)_3_	1.06/1.04/1.07
	V_sub_ = 937.7		V_prod_ = 990.4/973.7/1005.2	
(5)	3CaO·Al_2_O_3_·3CaSO_4_·32H_2_O	→	3CaCO_3 (Cal/Ar/Vat)_ + 2Al(OH)_3_ + 3(CaSO_4_·2H_2_O)	0.57/0.55/0.58
	V_sub_ = 704.5		V_prod_ = 398.6/390.3/406.1	

## Data Availability

The original contributions presented in this study are included in the article. Further inquiries can be directed to the corresponding authors.
